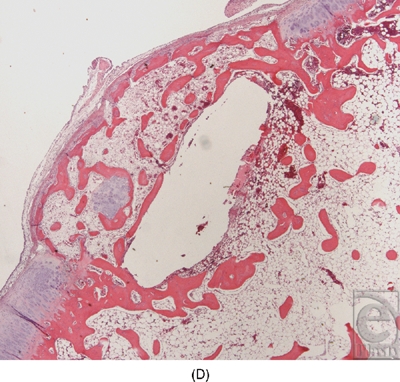# Use of a Smooth, Resorbable Template for Delivery of Cultured Pellets of Autologous Chondrocytes to Articular Cartilage Defects—Preliminary Report

**Published:** 2009-08-28

**Authors:** Bohdan Pomahac, Baraa Zuhaili, Yusef Kudsi, Pejman Aflaki, Elof Eriksson

**Affiliations:** Division of Plastic Surgery, Brigham and Women's Hospital, Harvard Medical School, Boston, MA 02115

## Abstract

**Background:** Autologous chondrocyte transplantation (ACT) is the most commonly used cell-based surgical procedure for repair of articular cartilage defects. The challenges of this technique include dedifferentiation of chondrocytes following several in vitro passages, invasive means of transplantation, and inadequate cell retention leading to washout of transplanted cells. To overcome these obstacles, we developed a novel technique of transplanting high-density chondrocyte pellets seeded on a prefabricated, resorbable, rigid, 2-dimensional template amenable to minimally invasive implantation. **Methods:** Chondrocytes were obtained from the costal cartilage of New Zealand white rabbits and expanded in vitro in monolayer culture. After 2 passages, chondrocyte suspension was centrifuged and a total of 1 × 10^6^ cells condensed on the surface of a prefabricated, resorbable template of LactoSorb plate (0.5-mm thick, 4-mm diameter). The construct was incubated for 24 hours in a culture medium before transplantation into circular 4-mm diameter, 0.5-mm deep defects in a non–weight-bearing part of the femoral condyle. Control defects were left empty or implanted with LactoSorb alone. Macroscopic and histological evaluation was performed 4 weeks posttransplantation. **Results:** Macroscopically, boundaries of all defects were demarcated and distinguishable from adjacent intact cartilage. Regenerative tissue in experimental group appeared white, smooth, and uniform showing more resemblance to hyaline cartilage. Control groups revealed absent cartilaginous tissue and defects were filled with soft, fibrous tissue with an irregular surface. Histologically, the repair tissue in the control groups was fibroinflammatory with irregular surface and no evidence of continuous chondrocytic regeneration. Cartilage regeneration in the experimental defects revealed a continuous, high-density layer of chondrocytes surrounding the LactoSorb plates. Consistently with chondrocyte pellets grown for 4 weeks only, the amount of extracellular matrix deposition in the transplanted group was less than the normal cartilage. **Conclusion:** We have developed a novel approach for ACT, utilizing high-density chondrocyte pellets seeded on a prefabricated, rigid, 2-dimensional resorbable carrier. Our study can serve as a model for further minimally invasive development of this technique and evaluating its potential role as an alternative in ACT.

Articular cartilage defects have limited regenerative capacity. Inadequate repair of articular cartilage defects after traumatic injury or in degenerative conditions may lead to osteoarthritis.^1^,^2^ Full-thickness cartilage injuries penetrating the subchondral bone lead to the migration of chondroprogenitor cells to the site of injury and formation of a stable fibrocartilage repair tissue. On the other hand, the reparative process in partial-thickness cartilage injuries, confined to the avascular cartilage layer, is poor because of the inability of the bone marrow cells to enter the defect and the limited migration and contribution of the neighboring chondrocytes to repair the cartilage defect.^3^,^4^

Autologous chondrocyte transplantation (ACT) is the most common cell-based procedure for repair of cartilage defects. The most widely used approach to this procedure involves transplantation of culture-expanded autologous chondrocyte suspension into a reservoir created by fixing a periosteal flap or collagen sheet to the surrounding cartilage. It has produced hyaline-like repair tissue in experimental models^5^–^7^ and achieved a widespread clinical use.^8^–^13^ However, obtaining sufficient number of differentiated chondrocytes (eg, [2–5] × 10^6^ cells) 5,7,8 is a major problem because these cells dedifferentiate rapidly during multiple passages in monolayer cultures.^14^–^17^ Other challenges include inefficient cell retention leading to loss of cells into the joint space, limitations in defect size and geometry, the need for an intact cartilage rim adjacent to the defect, disruption of the adjacent cartilage as a result of suturing the periosteal flap,^5^ and invasive implantation of chondrocytes under a periosteal flap requiring open knee operation with major postoperative morbidity. Similarly, perhaps because of microenvironment changes in pH during resorbable scaffold hydrolysis, chondrocytes seeded in porous matrices have undergone dedifferentiation with loss of shape and phenotype.^18^

High-density cultures have been widely used as in vitro model to study embryonic cartilage formation. By supporting cell-cell interactions, high-density cultures have been shown to promote chondrogenic differentiation.^19^,^20^ Dedifferentiated chondrocytes from monolayer passages (P1–P4) have been shown to regain their chondrocyte phenotype and form cartilage nodules when grown in pellets.^21^ In the pellet, the close spatial relationship of neighboring chondrocytes provides a better environment for cell-cell and cell-matrix communication, maintaining chondrocyte differentiation.^22^,^23^

To address several problems associated with autologous chondrocyte implantation, we designed a technique suitable for minimally invasive approach, allowing for the implantation of autologous chondrocyte pellets seeded on, or encapsulated in, a rigid, smooth, and resorbable carrier of poly-L-lactic acid (PLLA) and polyglycolic acid (PGA), commercially available as LactoSorb. We have previously shown that cartilaginous constructs enclosed in a resorbable template of LactoSorb retain their viability and shape.^24^ The current study evaluates the feasibility of LactoSorb as a carrier of high-density pellets of chondrocytes for transplantation of cultured autologous chondrocytes on LactoSorb molded to fit the size and shape of the chondral defect. The resorbable fixation systems have been clinically available since 1996 and extensively utilized in pediatric craniofacial fixation.^25^ Their basic composition consists of a copolymer of PLLA and PGA, which is degraded by hydrolysis into lactic acid and glycolic acid.^26^,^27^ Commercially available resorbable materials MacroPore (MacroPore Biosurgery, Inc, Kensey Nash, Exton, Pa), PolyMax (Synthes, Westchester, Pa), and LactoSorb (Biomet, Inc, Warsaw, Ind) have numerous compositions, differing mostly by the degradation speed. We selected LactoSorb because of the shortest resorption time of less than 12 months.

## MATERIALS AND METHODS

### Animals

All animal procedures were approved by the Harvard Medical Area Standing Committee on Animals. Six-month-old, skeletally matured New Zealand white rabbits weighing 3 kg were allowed to acclimatize for 1 week before starting the experiment. They were kept separately in smooth-walled stainless-steel cages, 1 animal per cage, with food and water ad libitum.

## Harvesting and culturing the chondrocytes

We obtained the chondrocytes from the cartilaginous parts of ribs 5 to 8. Skeletally mature New Zealand white rabbits were anesthetized using intramuscular injection of xylazine (5 mg/kg of body weight) and ketamine (45 mg/kg of body weight). Costal cartilage was chosen as a source of chondrocytes for 2 reasons: abundance of released cells leading to shorter culture time and to avoid violence of articular cartilage prior to the chondrocyte transplantation. After prepping and draping the anterior chest in a sterile fashion, midsternal skin incision was performed and the right pectoralis muscle was released from its sternal origin and reflected laterally to expose the costal cartilages. Meticulous attention was paid not to damage the parietal pleura. Hemostasis was achieved and incision was closed in layers.

After removing the perichondrium under sterile conditions, the harvested cartilage was sliced into 1-mm pieces, washed 3 times in sterile phosphate-buffered saline containing 100 units/mL of penicillin, 100 µg/mL of streptomycin (GIBCO; Invitrogen, Grand Island, NY), and 0.25 µg/mL of amphotericin B (Bristol-Myers Squibb, Princeton, NJ), and digested for 18 hours in 15 mL of Dulbecco's Modified Eagle's Medium (DMEM) containing 0.2% collagenase II (Worthington Biochemical, Freehold, NJ) and antibiotics (as above) at 37°C in a 5% CO_2_ incubator. Subsequently, 20 mL of DMEM was added and the resulting chondrocyte suspension was homogenously distributed by repeated pipetting for 10 times. The liberated cells were then passed through a sterile 250-µm nylon mesh filter and collected by centrifugation at 1200 rpm for 6 minutes. Ham's F-12 medium (Hyclone; Fischer Scientific, Pittsberg, Pa) supplemented with 10% fetal bovine serum, 100 units/mL of penicillin, 100 µg/mL of streptomycin, 0.25 µg/mL of amphotericin B (Bristol-Myers Squibb), 50 µg/mL of L-ascorbic acid 2-phosphate Mg (Sigma-Aldrich, St Louis, Mo), 2 mM of L-glutamine (Sigma-Aldrich), 1% nonessential amino acids (Sigma-Aldrich), and 5 µg of TGF-*β*1 (Sigma-Aldrich) was used for cell culture. The recovered chondrocytes were resuspended in 20 mL of culture medium and homogenized by repeated pipetting for 20 times. An aliquot of the suspension was exposed to trypan blue (Sigma-Aldrich) to determine cell viability, and those cells that excluded the dye were counted with a hemocytometer. The resulting cell suspension was cultured in 100-mm dishes (FALCON; Becton Dickinson Labware, Wyckoff, NJ) at 37°C in a 5% CO_2_ incubator. The medium was changed on day 1 and every 4 days afterward. Upon reaching confluence, 1 × 1^6^ chondrocytes were condensed by centrifugation at 1000 rpm for 5 minutes, forming high-density pellets of chondrocytes, which were then seeded on LactoSorb and incubated in the culture medium prior to implantation.

**Figure 1:**
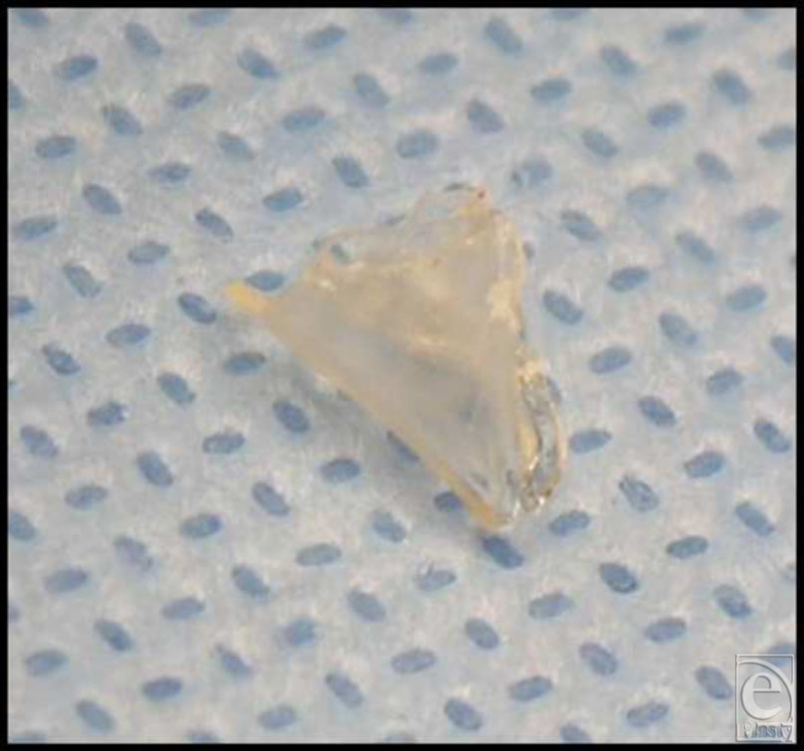
A cone made from resorbable carrier (A) is used to carry the chondrocyte pellets to deep cartilage defects (B).

This study was conducted in 2 parts. In the first set of experiments, high-density pellets of chondrocytes were seeded onto LactoSorb, molded as a cone (Fig 1A), and the resulting construct was incubated for 6 weeks during which time, individual chondrocytes gained organized structure resembling hyaline cartilage. The construct was then implanted into the deep cartilage defect (Fig 1B). Rabbits were euthanized after 2 weeks to evaluate the cartilage repair. This part was done to evaluate the ability of chondrocyte pellets to maintain their phenotype both in vitro and in vivo after prolonged culturing and to assess the LactoSorb potential as efficient carriers of chondrocytes.

**Figure 2:**
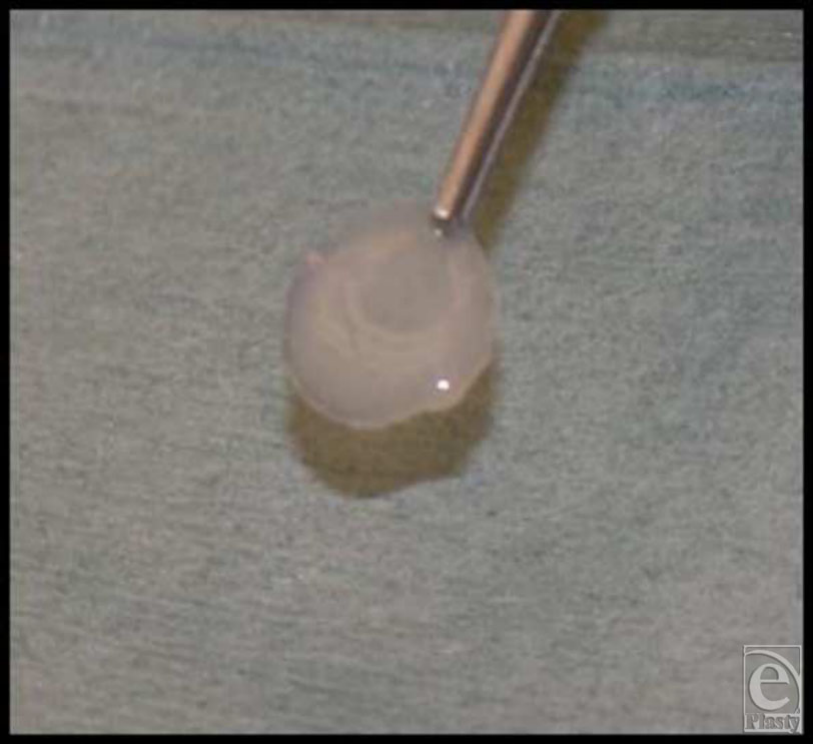
Demonstration of the LactoSorb plates of 0.5-mm thickness and 4-mm diameter before seeding the chondrocyte pellets on its surface.

In the second part of the study, high-density pellets of chondrocytes were seeded on round LactoSorb plates of 0.5-mm thickness and 4-mm diameter (Fig 2) and incubated for only 24 hours in the culture medium before transplantation into 0.5-mm deep cartilaginous defects. This way, transplanted chondrocytes were directly facing the bed of the defect. In this latter part of the experiments, cells were passed twice before they were condensed at a density of 1 × 10^6^ onto the surface of LactoSorb plates. Here, the assessment of repair tissue was done 4 weeks after transplantation. In all experiments, LactoSorb plates were coated with poly-L-lysin to negatively charge its surface and improve adherence of cells.

## Creation of defect and chondrocyte transplantation

**Figure 3:**
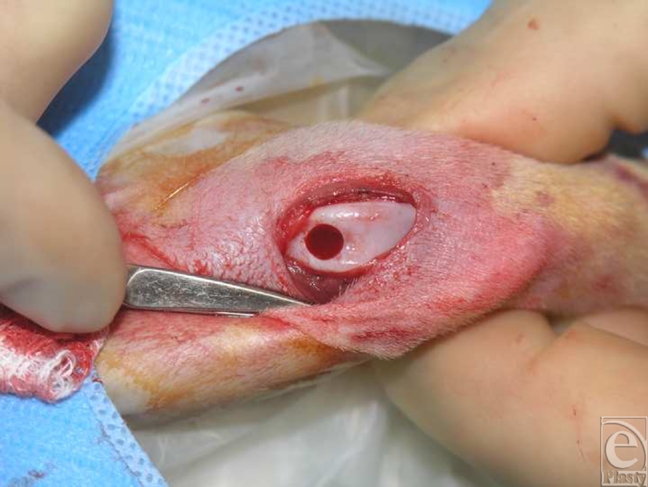
A 4-mm diameter circular defect was created in the patellar groove of the distal femur down to subchondral bone with a stainless steel punch. The depth was gauged by a mark on the punch device at 0.5 mm, allowing smooth contouring of the joint by the added thickness of the LactoSorb.

General anesthesia was induced as described. The animals were placed supine on the operating table and the experimental knee was shaved, prepared, and draped in a sterile fashion. The knee was entered by a longitudinal medial parapatellar incision and the patella was dislocated laterally to expose the articular surface of the femoral condyles. A circular defect, 4-mm in diameter, was created in the patellar groove of the distal femur down to subchondral bone with a stainless steel punch (Fig 3). The depth was gauged by a mark on the punch device at 0.5 mm, allowing smooth contouring of the joint by the added thickness of the LactoSorb. The LactoSorb pellet with cultured chondrocytes on its surface facing the defect was then implanted. In our experience, the repositioned patella provides adequate fixation of the carrier in desired position and therefore additional fixation is not necessary. Incision was closed in a layered fashion. Each rabbit had only 1 knee operated, and a total of 30 defects were created in 30 rabbits. Our groups were composed of a transplanted group that received chondrocyte-seeded LactoSorb (*n* = 10) and 2 control groups, in which defects were either left empty (*n* = 6) or filled with LactoSorb alone (*n* = 6). The patella was then reduced and the wound was closed in layers using 5-0 vicryl interrupted sutures. Postoperatively, the rabbits were returned to their cages and allowed to ambulate freely.

## Evaluation of reparative tissue

Four weeks after transplantation, the animals were euthanized by an overdose injection of pentobarbital and their chondral defect was inspected for shape, contour, and integration of reparative tissue with adjacent native cartilage. The specimens were then fixed with paraformaldehyde, decalcified overnight, embedded in paraffin, and sectioned for hematoxylin and eosin (H&E) staining.

## RESULTS

**Figure 4:**
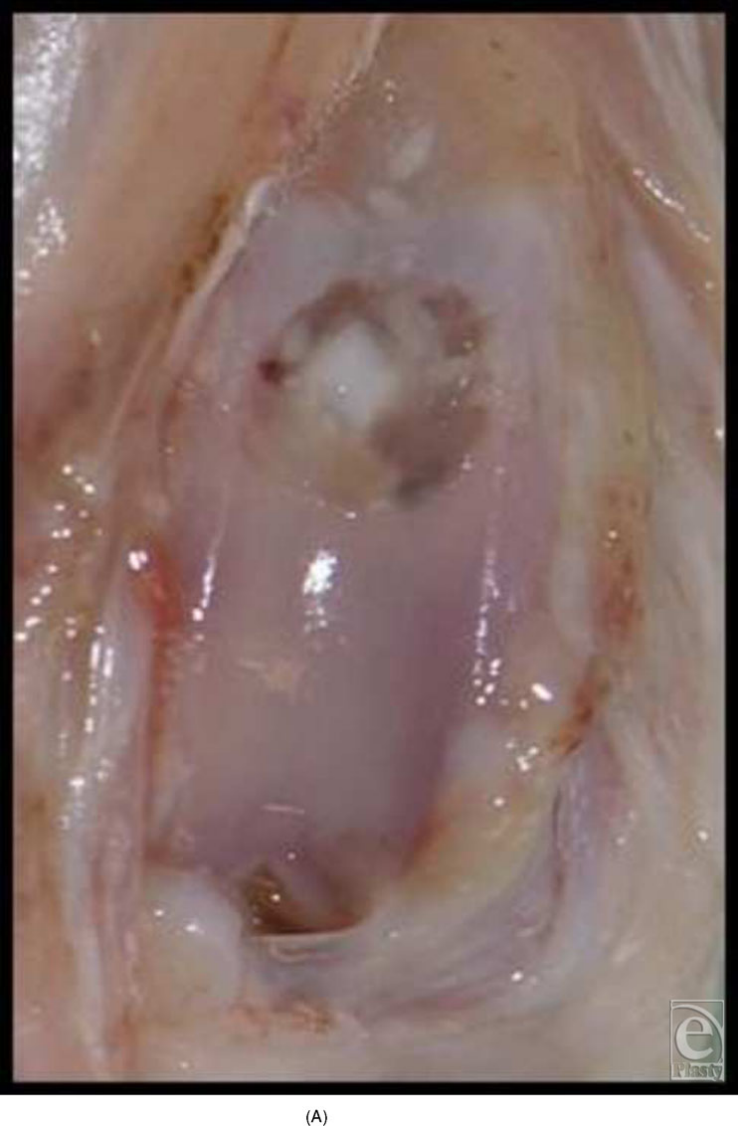
Knee joint 2 weeks following transplantation of chondrocyte pellet in a cone of LactoSorb reveals hyaline-like cartilage (A) confirmed histologically (B). The central white area corresponds to the transplanted chondrocyte pellet. Unhealed control defect at 2 weeks, with partial fibrous repair demonstrated in part C.

In the early experiments with implantation of cone-shaped LactoSorb constructs containing chondrocyte pellets, chondrocytes seemed to redifferentiate and maintained their chondrogenic potential for 8 weeks (6 weeks in vitro culturing and 2 weeks in vivo), evidenced by the formation of hyaline-like cartilage both in gross appearance and in histological sections in contrast to the control defects (Fig 4).

**Figure 5:**
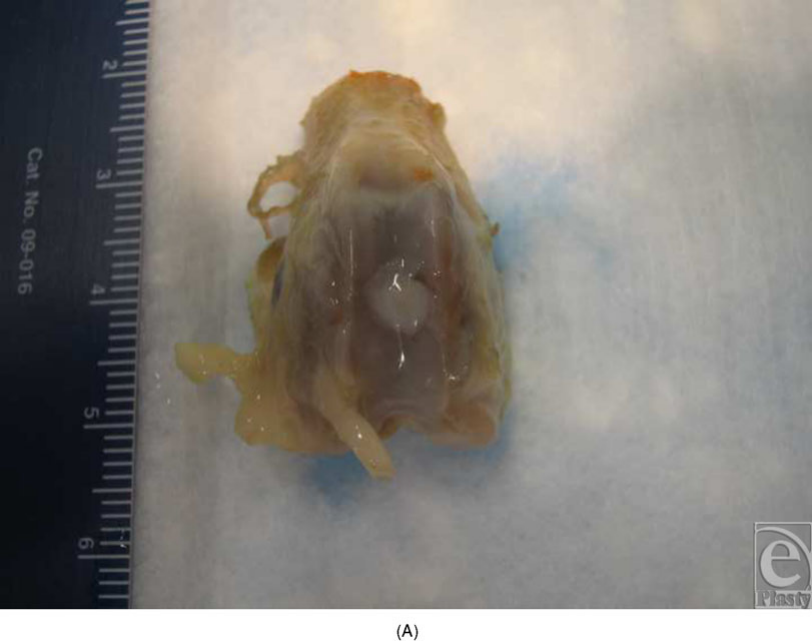
Macroscopic appearance of the repair tissue in the transplanted group (A) appeared white, smooth, and uniform in texture showing more resemblance to healthy normal hyaline cartilage. In the control groups (B), cartilaginous tissue was generally absent and the defects were filled with nonspecific soft, fibrous tissue with an irregular surface.

**Figure 6:**
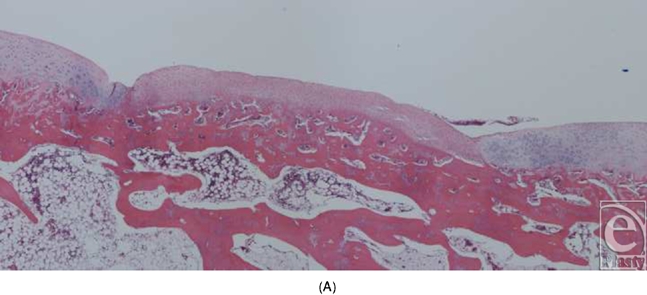
Histological section of the defects 4 weeks after creation. The empty defects (A and B) are filled with fibrous repair, with absence of continuous chondrocytic layer bridging the defect. In a representative experimental defect (C), newly formed cartilage is demonstrated by a thick and continuous layer of chondrocytes underneath the LactoSorb plate, suggesting its origin from the transplanted pellet. There is also a continuous layer of chondrocytes above the LactoSorb plate, likely resulting from migration of transplanted chondrocytes. The untransplanted control groups (LactoSorb alone with no chondrocytes) are shown in part D. Here, the defect is filled with fibrovascular tissue and there is no evidence of cartilaginous regeneration.

In the experiments in which round pieces of LactoSorb plates were used as a platform for high-density seeding of chondrocytes, macroscopic examination revealed that the boundaries of defects in all knees were demarcated and distinguishable from the adjacent intact cartilage. In general, the regenerative tissue in the transplanted group appeared white, smooth, and uniform in texture showing more resemblance to healthy normal hyaline cartilage (Fig 5A). In the control groups, cartilaginous tissue was generally absent and the defects were filled with nonspecific soft, fibrous tissue with an irregular surface (Fig 5B). Histologically, healing was evaluated by cellular morphology and the presence of a continuous layer of chondrocytes filling the defect. At 4 weeks, the chondral defects in the control groups were filled with fibrous tissue and none of them showed evidence of full chondrocytic regeneration. Cellular morphology was described as fibrous and inflammatory in nature (Fig 6A). Surface was not continuously covered with chondrocytes and was irregular. Cartilage regeneration in the experimental defects was significantly superior compared with that of the control defects revealing a continuous layer of chondrocytes surrounding the LactoSorb plates the majority of the time, indirectly confirming its origin from the transplanted pellet (Fig 6B). Consistently with chondrocyte pellets grown for 4 weeks only, the amount of extracellular matrix deposition in the transplanted group was grossly less than the normal cartilage.

## DISCUSSION

Articular cartilage has limited capacity for spontaneous regeneration. Inadequate repair leads to pain and disability and may result in degenerative arthritis.^1^,^2^ Following limited and short-term success of various marrow-stimulating surgical procedures^28^–^32^ in promoting fibrocartilaginous repair in focal cartilage defects and their inferior biomechanical properties, cell-based treatment modalities were developed to achieve a more durable and functional cartilage. Autologous chondrocyte transplantation in the treatment of focal articular cartilage defects gained popularity in the early 1990s and has achieved a widespread clinical use.^8^–^13^ Following in vitro expansion, the chondrocyte suspension is injected into a pocket created by suturing a periosteal flap to the surrounding cartilage. Acquisition of sufficient number of chondrocytes that can maintain their differentiated phenotype is a major challenge for this technique and remains unresolved. In addition, it requires an open knee operation that is associated with major morbidity and lengthy postoperative rehabilitation. Wide exposure necessary for performing this procedure is one of the major reasons why this technique has been limited only to the knee joint.

Despite advance in cartilage tissue engineering and 3-dimensional matrices, the search for an optimal method to enhance cartilage regeneration continues. In this study, we evaluated the feasibility of autologous chondrocyte pellets as a regenerative source in the treatment of focal cartilage defects. High-density cultures promote chondrogenic differentiation of multipotential mesenchymal cells^19^,^20^ and enhance chondrocyte phenotype after multiple passages (P1–P4) in prolonged monolayer chondrocyte culturing.^21^ In the pellet, the close spatial relationship of neighboring chondrocytes provides a better environment for cell-cell and cell-matrix communication, maintaining chondrocyte differentiation.^22^,^23^ To deliver the chondrocyte pellets in vivo to focal articular cartilage defects, we used a novel technique, in which 1 × 10^6^ chondrocytes were condensed on the surface of a resorbable carrier (LactoSorb) that was trimmed to the shape and size of the cartilaginous defect prior to implantation. We observed superior cartilage regeneration in the defects with transplanted chondrocytes compared with the untreated defects.

This publication reports our preliminary experience with transplantation of high-density chondrocytes seeded on resorbable templates of PLLA and PGA—LactoSorb—to focal articular defects. Our approach has several advantages. Although we used an open technique for our experimental transplantation, our technique has minimal-invasive applicability; the implantation of the plate could be accomplished by arthroscopic or arthroscopy-assisted techniques, reducing operating time and postoperative rehabilitation. One of the major challenges of the commonly used ACT procedures is the dedifferentiation of chondrocytes during multiple-passage monolayer culturing, which is needed to obtain sufficient numbers of chondrocytes to fill the cartilaginous defects. By promoting redifferentiation of chondrocytes and enhancing their chondrogenic potential, our technique of transplanting pellets instead of chondrocytic suspensions allows for adequate expansion of cells prior to transplantation. In addition, our delivery method of using rigid, smooth, and resorbable LactoSorb templates that are prefabricated to fit the cartilaginous defect dimensions, with the chondrocyte pellets seeded on its surface, can increase the retention of cells and minimize their washout, which seems to be a disadvantage of transplanting chondrocyte suspensions. In the future, the shape and size of the cartilaginous defect could be obtained from 3-dimensional computed tomographic scan, magnetic resonance imaging, or arthroscopic imprint and chondrocyte-seeded carrier manufactured correspondingly. Furthermore, the rigidity of this carrier system allows for transmission of mechanical forces on the transplanted chondrocytes that have been found important for their chondrogenic phenotype.^33^–^36^ It can also be fixed with resorbable screws if needed. Ultimately, the most useful application of this technique would be a 1-stage harvest and implantation of the autologous chondrocytes. This advancement would eliminate 1 procedure in the operating room and the regulatory burden for off-site cell-culturing facilities.

This article reported our novel approach to ACT. We tested the suitability of our carrier system in delivering high-density chondrocyte pellets to cartilage defects. We have no intention of claiming superiority of our technique over other methods of ACT because a more detailed and longer term assessment of its histological and functional outcomes is required. The limitations of this study included a lack of quantitative analysis of the repair tissue and a relatively short follow-up. However, our study can serve as a model for further developments of this technique and evaluating its potential role as an alternative in ACT.

## Figures and Tables

**Figure:**
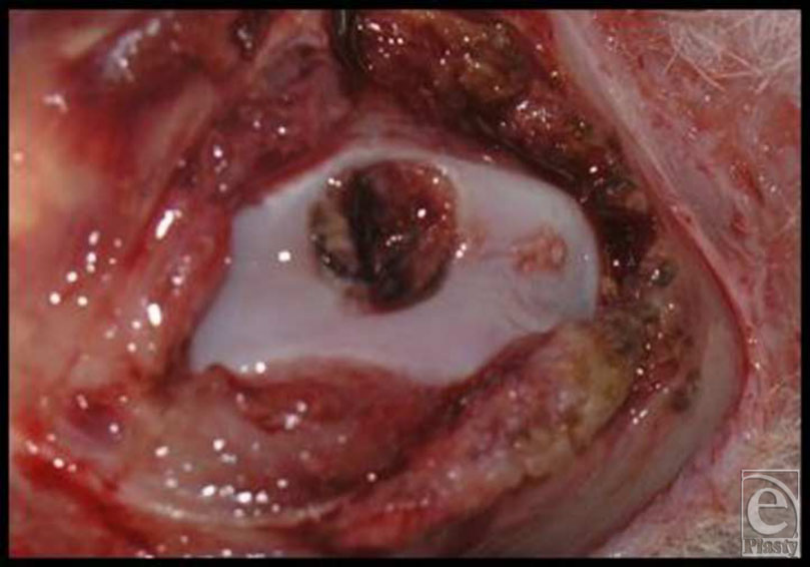


**Figure:**
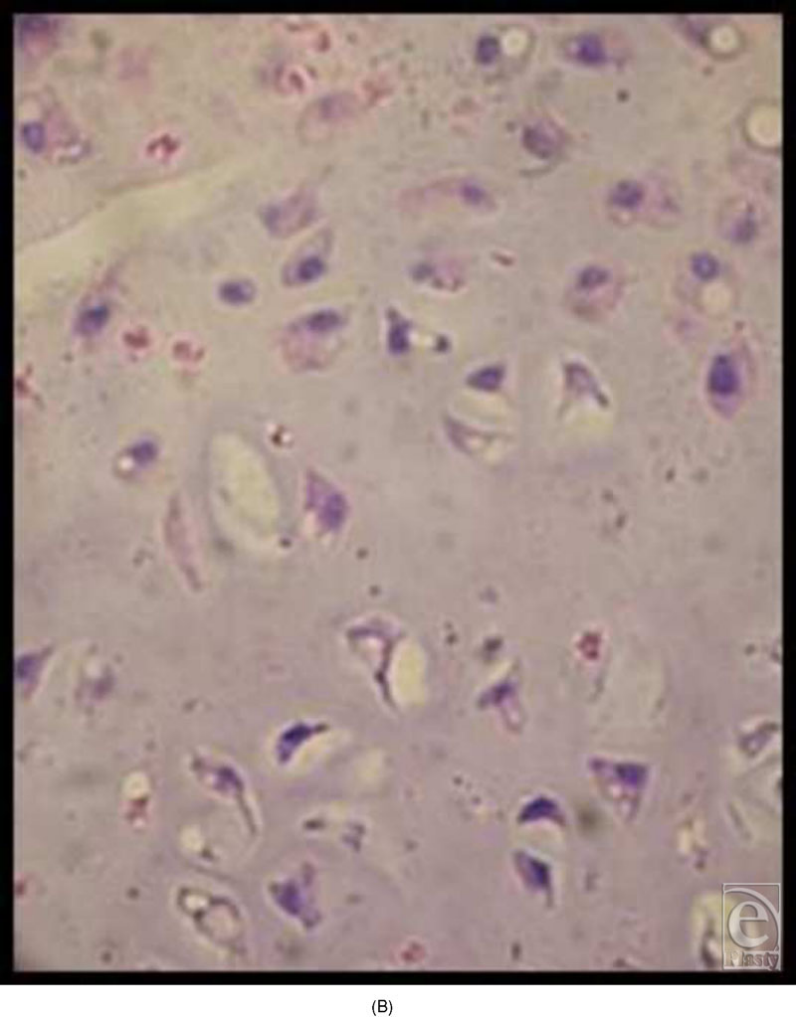


**Figure:**
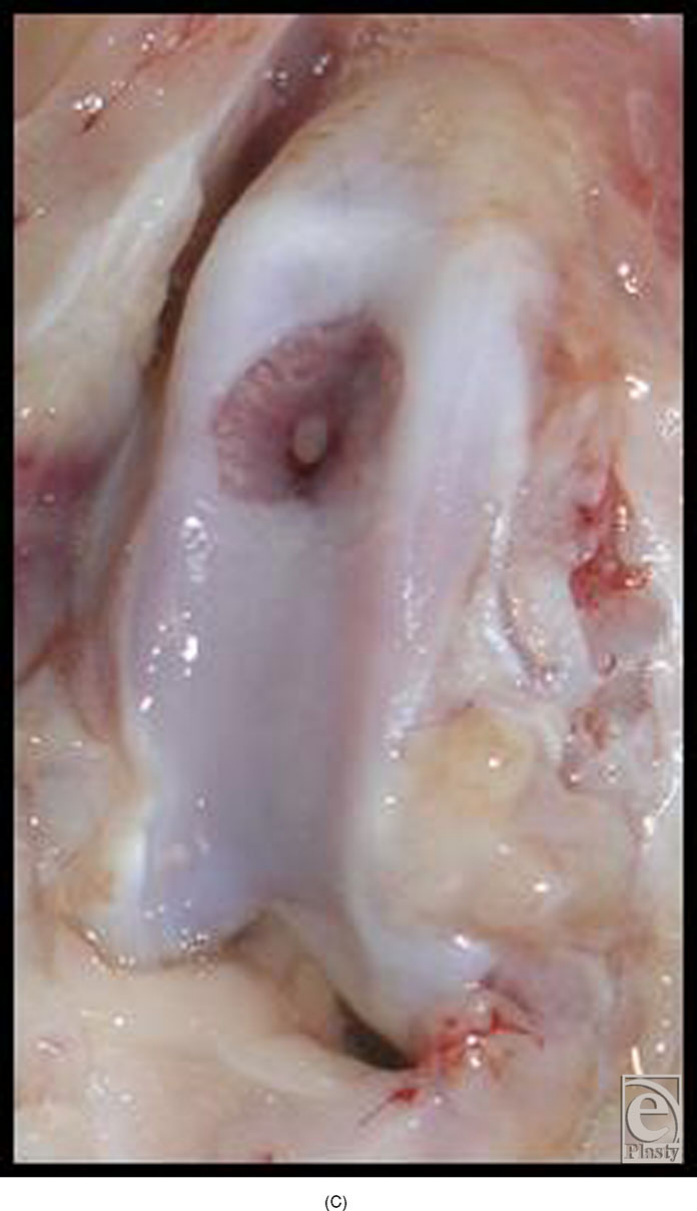


**Figure:**
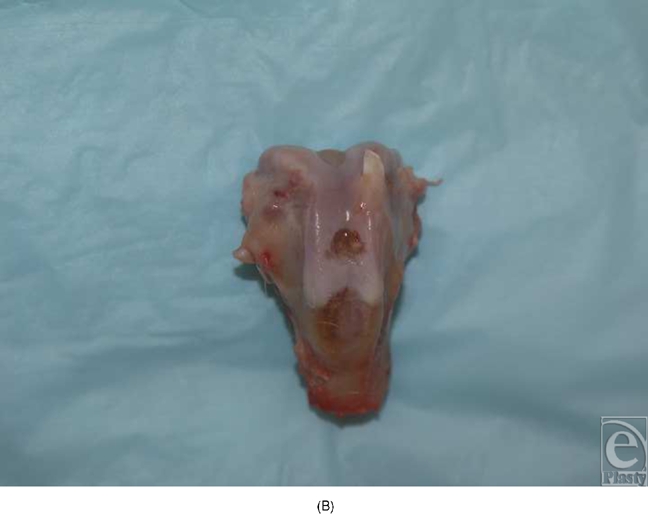


**Figure:**
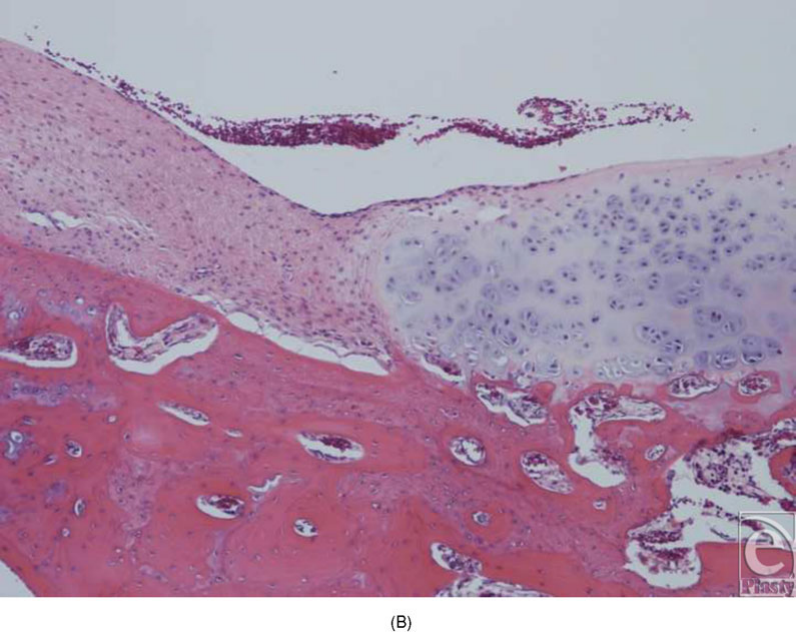


**Figure:**
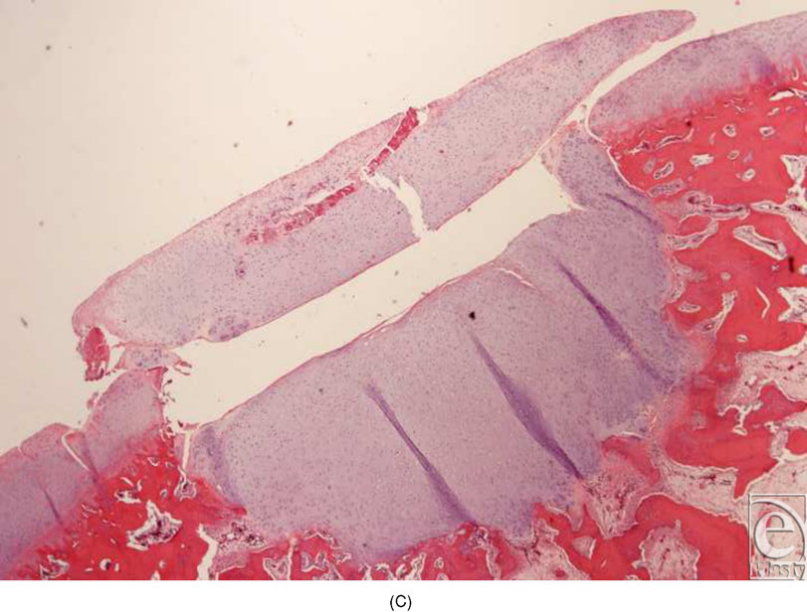


**Figure:**